# Optimizing MEMS inertial switches for efficient event-based environmental interaction: motivation, approaches, and purposes

**DOI:** 10.1038/s41378-025-00997-1

**Published:** 2025-08-12

**Authors:** Lyuyan Wang, Jiahao Zhao, Zheng You

**Affiliations:** 1 Department of Precision Instrument, Tsinghua University, Beijing, 100084 PR China; 2 Key Laboratory of Smart Microsystem, Ministry of Education, Beijing, 100084 PR China; 3 State Key Laboratory of Precision Measurement Technology and Instruments, Tsinghua University, Beijing, 100084 PR China; 4 Beijing Advanced Innovation Center for Integrated Circuits, Beijing, 100084 PR China

**Keywords:** Sensors, Electrical and electronic engineering

## Abstract

The rapid growth of the Internet of Things (IoT) and embodied intelligence has increased the demand for sensor nodes that conserve energy and reduce data transmission, especially in resource-limited applications that rely heavily on sensors. Event-based sensors have emerged to meet this demand by reducing data redundancy and lowering power consumption. Within this domain, MEMS (Micro-Electro-Mechanical Systems) inertial switches stand out as promising alternatives to traditional commercial accelerometers and gyroscopes, catering to the widespread need for inertial sensing. This review categorizes the key aspects for optimizing the performance of MEMS inertial switches, with a focus on threshold sensitivity, directional responsiveness, and contact performance. It explores the technological pathways for achieving these objectives and highlights the wide-ranging applications of MEMS inertial switches, especially in scenarios characterized by energy constraints, large-scale deployments, and harsh environments. Additionally, the current challenges faced in the field are analyzed, and future research directions are proposed to enhance the versatility and integration of MEMS inertial switches, thereby promoting their broader adoption and utility.

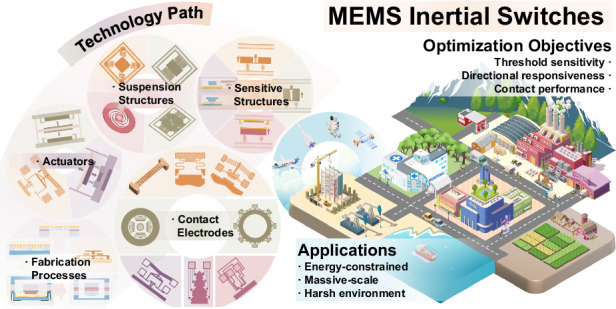

## Introduction

Sensing technology has revolutionized our interaction with the physical world, spurring widespread demand for real-time sensor networks across industries. These sensors are essential for the growth of the Internet of Things (IoT), which is projected to encompass 40 billion devices by 2030 (ref. ^1^). They also support embodied intelligence—autonomous systems integrating multimodal sensor data to dynamically perceive, interpret, and respond to physical environments^2^. While high-performance sensors are indispensable for enabling rapid responses and intelligent decision-making in IoT and embodied intelligence, their scalability faces a fundamental barrier: the unsustainable energy and computational demands of supporting an ever-growing network of sensor nodes^3–6^.

Traditional battery-powered sensors, operating in continuous active modes, exhibit severe limitations in remote or hard-to-reach locations and in sensor-intensive but resource-limited embodied intelligence terminals, where battery replacement is impractical or costly. High-frequency sampling strategies in such sensors further compound inefficiencies by generating substantial volumes of redundant data since the target events occur sporadically^7^. This redundancy critically degrades the energy and computational efficiency, especially in Artificial Intelligence (AI) systems during training and inference, contrasting starkly with the efficiency-optimized sensory processing in biological systems^8,9^.

Event-based sensing has emerged as a transformative approach to address these limitations. The Near Zero Power RF and Sensor Operations (N-ZERO) program^4^ exemplifies this paradigm, enabling sensors to maintain low-power sleep states while activating only upon detecting predefined physical, chemical, or radio frequency (RF) signatures^4^. This strategy minimizes reliance on energy-intensive continuous data processing algorithms^6^. Neuromorphic computing advancements complement this approach by emulating biological neural architectures to develop energy-efficient processors. However, this technology faces compatibility barriers with traditional frame-based data formats—dense synchronous arrays contradict the event-driven nature of neuromorphic hardware^10^. Large-scale neuromorphic computing, therefore, requires sensor redesigns adopting an event-based paradigm, which achieves dual improvements in power efficiency and latency^6,10^. These event-driven technologies generate sparse temporal coding, preventing low-power interfaces from being overwhelmed by unnecessary data. This co-design bypasses redundant data conversion steps, conserves power, memory, and postprocessing resources, and offers beneficial features such as self-adaptation, low latency, and high dynamic range^10,11^.

The MEMS wake-up switch exemplifies event-based sensing with three core attributes: micrometer-scale footprint, nanowatt-scale static power^4^, and multimodal functionality as an integrated sensor-processor-actuator. These devices maintain a zero-power static state while monitoring environmental inputs, generating spike/step signals exclusively upon detecting threshold-exceeding events. The output signals serve dual purposes: (1) controlling downstream circuits or systems for event recording, critical data transmission, or activating more comprehensive sensing and actuation systems, and (2) encoding precise timing of spikes or a sequence of spikes compatible with Spiking Neural Networks (SNNs)^12–14^. This methodology captures only essential data, boosting system intelligence and efficiency^15,16^. Moreover, biology shows that the event-based paradigm applies not just to perception and inference but also to control^14^. Therefore, integrating these switches into the low-level modules of embodied intelligence can facilitate partial autonomy and amortized control. This allows low-level modules to operate semi-autonomously during inactivity from high-level modules and to manage fast reflexive responses effectively^17^.

Inertial monitoring requirements are almost ubiquitous across various applications, serving multiple functions such as attitude control, motion monitoring, and environmental sensing. Within IoT networks and embodied intelligence devices, continuous inertial tracking enables real-time detection of abnormal impacts, vibrations, and motion states. This capability not only prevents equipment damage by triggering protective mechanisms but also captures event-specific system parameters for post-failure analysis and adaptive control algorithms development. MEMS inertial switches represent a category of MEMS wake-up switches designed for inertial sensitivity, delivering efficient and energy-saving solutions for these monitoring demands. Their ultralow energy profile permits resource redistribution to other working modules and prolongs battery life in continuous monitoring.

MEMS inertial switches offer unique advantages for IoT and embodied intelligence applications. In energy-limited settings (underwater systems^5^, border regions^18,19^, wearables^20^), their near-zero leakage currents and nanowatt-level operation enable maintenance-free sensor networks and prolong the lifetime of missile monitoring^21^, intrusion detection^18^, or fall-detection^22,23^ devices. For massive infrastructure, agriculture, and logistics deployments, their ultra-low latency and simplified circuitry support cable-free, cost-effective monitoring of time-sensitive events while minimizing network maintenance costs^10,15,24–26^. In extreme conditions (aerospace, defense, automotive), their radiation tolerance, electromagnetic interference immunity, and mechanical robustness ensure reliability for safety-critical tasks like airbag deployment or hazardous environment monitoring^8,27–30^. These attributes collectively address challenges of power efficiency, scalability, and operational resilience across diverse sectors.

This review provides a comprehensive overview and classification of MEMS inertial switches. We begin by summarizing the common design objectives for these switches, highlighting key features such as extremely high or low thresholds, multi-directional capabilities, adjustable or multi-threshold, and mechanisms for contact enhancement and self-latching. Next, we delve into how the specific design of sensitive structures, suspension structures, contact electrodes, actuators, and appropriate fabrication processes can effectively achieve these design objectives. We then outline the broad application prospects of MEMS inertial switches, highlighting their unique performance in practical technology, manufacturing, and everyday life. Finally, we discuss the current challenges and future outlook for MEMS inertial switches, providing insights into potential advancements in the field.

## Optimization objectives

MEMS inertial switch advancements demonstrate transformative potential across industrial applications. These switches are becoming more reliable, precise, and adaptable, designed to meet a vast array of operational conditions. Researchers have engineered multiple variants distinguished by acceleration thresholds, directional response, and contact performance. The optimization objectives for MEMS inertial switches can be categorized into seven critical parameters, as detailed in Table 1. These areas include expanding the threshold range to achieve extraordinarily high and low values, integrating multiple thresholds and adjustable thresholds, enabling multi-directional responsiveness, improving contact performance, and incorporating self-latching capabilities.

**Table 1:** Overview of the performance attributes of several typical MEMS inertial switches with optimization objectives of High-threshold (HT), Low-threshold (LT), Multi-threshold (MT), Tunable-threshold (TT), Multiple-directional (MD), Contact-enhanced (CE), and Self-latching (SL)

Optimization objectives	Threshold (g)	Contact time (μs)	Directional sensitivity	Characteristic	Ref
*Unoptimized*	100	12	uniaxial	-	^59^
HT, SL	4600	∞	uniaxial	Cylindrical contacts and an easy-latching/difficult-releasing latching mechanism.	^36^
HT, SL	15000	∞	uniaxial	Open and close paths separated from the proof mass.	^34^
HT, SL	3500	∞	uniaxial	An angled latching mechanism is used to avoid de-latching the switch.	^27^
HT, MT, SL	3000–15000	∞	uniaxial	A zigzag slot within the mass for acceleration load recognition	^32^
LT	5	-	uniaxial	A large proof mass supported by circular spiral springs.	^29^
LT, MT, SL	1.9–9.7	∞	uniaxial	A ratcheting mechanism to discretize measured amplitude with a 6-bit resolution.	^51^
LT, TT, SL	0–1	∞	uniaxial	Digitally operated 2-bit tunable MEMS accelerometer with electrostatic actuators.	^40^
LT, TT, SL	3.75–6	∞	uniaxial	Combines permanent magnets, ferromagnets, and flat coils to achieve latching and adjust the threshold.	^65^
LT, MD, CE	4.5–6	112–366	three-dimensionalomnidirectional	Uniform Archimedean spiral beams, equipped with flexible contact electrodes, serve to lower system stiffness and improve spatial threshold consistency.	^96^
LT, MD, CE	7.9–11.3	238–440	two-dimensionalomnidirectional	Four radial electrodes and one axial electrode were used to determine the acceleration direction by detecting closed states.	^46^
MT, TT	0–1113, 0–1600	10–110	uniaxial	Two fixed electrodes enable multi-threshold sensing, with parallel-plate electrodes providing tunable threshold options.	^47^
MT, MD	1197, 2186, 3314	10–170	biaxial	Three stationary electrodes with different distances to the movable electrode.	^43^
MT, SL	20–250	∞	uniaxial	Reusable threshold shock sensor with ten threshold levels.	^52^
TT, CE	20	540	uniaxial	Electrostatic force assistance and multi-step pulling action.	^76^
MD, CE	350–400	40–120	three-dimensionalomnidirectional	Combines a single-proof mass with flexible electrodes for an omnidirectional switch. Flexible electrodes prolong contact time, ensuring reliable signals.	^102^
CE	19–78	1100–4800	uniaxial	Gallium-indium (EGaIn) is adopted as the switching metal droplet.	^83^
CE	53	6000	uniaxial	The squeeze film effect can prolong the ON-state duration.	^30^
CE	38	114	uniaxial	Carbon nanotube (CNT) contact pads can prolong contact time.	^62^

### Ultra-High/Low thresholds

**Fig. 1:**
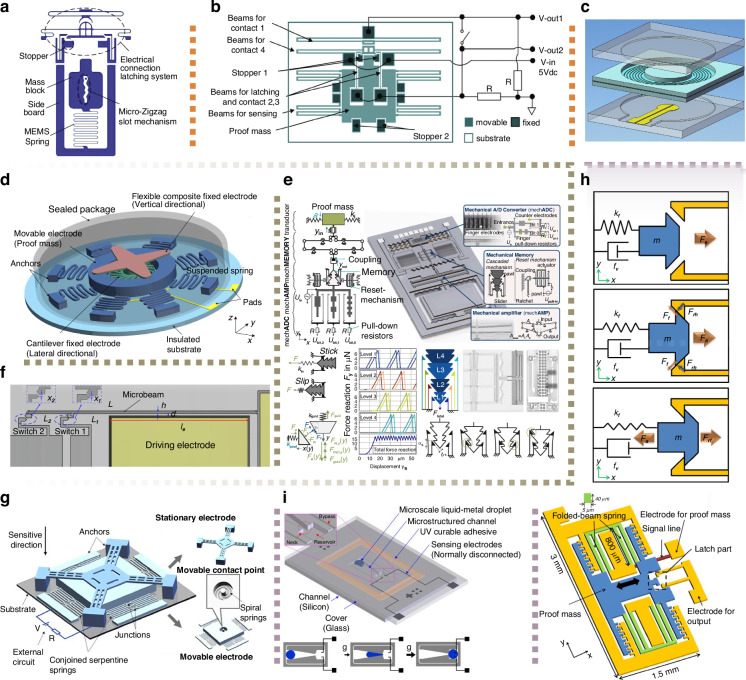
Design Objectives of MEMS inertial switches. **a** A fuse-integrated micro-switch featuring a zigzag slot design can differentiate a 3000 g smooth launch from a 15000 g accidental fall^32^. **b** High-g latching switch (4500 g threshold) with meticulously crafted contact shapes and latching mechanisms to keep contacts closed under intense shocks^35^. **c** Low-g MEMS switch (5 g threshold) uses circular spiral springs and a double-buried SOI wafer to make the spring longer and thinner, reducing stiffness and achieving a low threshold^29^. **d** A multi-directional inertial microswitch with a uniform 70 g threshold in the xy-plane and z-axis, achieved with eight snake springs for even stiffness^44^. **e** A passive acceleration sensor uses a ratcheting mechanism to detect and store peak acceleration values with 6-bit resolution, measuring and recording amplitudes from 1.9 g to 9.7 g^51^. **f** A tunable MEMS inertial switch features a beam segment as one electrode. Applying a bias voltage generates electrostatic actuation, allowing adjustment of the threshold value^47^. **g** Compliant cantilever electrodes paired with a dual mass-spring movable electrode allow elastic deformation during contact, ensuring stable, prolonged electrode interactions^22^. **h** A MEMS acceleration switch with mechanical latching maintains a reliable on-state after experiencing accelerations exceeding 43.7 g^54^. **i** A configurable MEMS inertial switch using a microscale liquid-metal droplet in a microstructured channel^72^

High-threshold scenarios such as downhole drilling and smart ammunition systems demand accelerometers that withstand vibrations surpassing 10,000 g and intense gun-hard shocks^31^. MEMS technology demonstrates a significant intrinsic potential in terms of shock resistance. For instance, Nie et al.^32^ designed a fuse-integrated switch with zigzag slots (Fig. 1a) that can differentiate between smooth launch accelerations (3000 g/3 ms) and accidental fall events (15,000 g/0.3 ms), ensuring fail-safe power circuit activation via controlled inertial response. Guo et al.^33–37^ developed a series of high-g latching switches (4500–15,000 g range) using special contact geometries and latching mechanisms to minimize contact resistance and ensure that the contacts remain closed under high-acceleration shocks (Fig. 1b).

Low-threshold applications, like health monitoring devices and specialized industrial transportation systems, require inertial switches with activation thresholds below 30 g^38^. Modern MEMS technology has facilitated the development of compact, lightweight, and high-performing low-g switches^39^. For example, Zhang et al.^29^ attained 5 g detection thresholds through circular spiral spring geometry and double-buried layer silicon-on-insulator (SOI) wafer processing, strategically elongating springs while decreasing thickness (Fig. 1c). To further reduce the threshold, Kumar et al.^40^ employed parallel plate electrostatic actuators and reduced the gap between the proof mass and the metallic tip to 270 nm by depositing a thick layer of gold that extended to the side walls. This refined design allows for precise detection of an acceleration of 0.38 g. Overall, MEMS inertial switches with various threshold levels showcase their multifunctionality in extreme shock monitoring and precise acceleration sensing.

### Multi-directional, multi-level, and tunable thresholds

Reported inertial micro-switches predominantly feature single-threshold unidirectional detection, limiting deployment in complex scenarios like traumatic brain injury (TBI) monitoring, where multiaxial sensing is essential because impacts can occur from any direction^41,42^. Additionally, quantitative range identification (e.g., 100–150 g) provides more valuable information than binary threshold reporting (e.g., 60 g exceedance)^43^. While multi-switch arrays address coverage of threshold and direction, they lead to issues such as excessive size, reduced sensitivity, installation inaccuracies, and centroid deviation^44^. IoT applications further expose adaptability limitations, as single-threshold devices require application-specific redesigns and costly microfabrication iterations, increasing production costs and reducing efficiency.

Multi-directional MEMS inertial switches are more cost-effective and easier to integrate than unidirectional arrays because they can detect shock vibrations from multiple directions^45^. To evaluate their performance, key metrics include omnidirectional sensitivity, precise identification of load direction, and consistent threshold distribution^46^. Yang et al.^44^ demonstrated 70 g threshold uniformity within the xy-plane and along the z-axis via an eight-snake-springs configuration (Fig. 1d). Despite these advancements, three-dimensional omnidirectional MEMS inertial switches still face challenges in achieving consistent acceleration thresholds, primarily due to non-uniform spring stiffness across the hemisphere.

Multi-level MEMS inertial switches operate as mechanical analog-to-digital converters, quantifying accelerations through discrete thresholds while maintaining simplicity^43^. Multiple electrodes with incremental spacing^43,47–50^ and ratchet mechanisms with bistable positions^51–53^ represent two methodologies enabling multi-threshold operation. An example is the passive acceleration sensor developed by Schmitt et al.^51^, which uses a cascaded ratcheting mechanism as mechanical memory to detect peak acceleration values (Fig. 1e). This sensor achieves a 6-bit resolution across 1.9–9.7 g. Currently, the design and performance of multi-threshold inertial switches are in the early stages of development, with ongoing research focused on enhancing working performance, safety, reliability, and system integration^15^.

Tunable-threshold MEMS inertial switches enable real-time adaptation across dynamic operational environments. The electrostatic softening effect provides a well-established method for adjusting trigger thresholds^40,47,50,54,55^. Xu et al.^47^ demonstrated this approach using a cantilever beam and parallel-plate electrode configuration (Fig. 1f), achieving an 85–1085 g threshold range via 0–22.5 V actuation. Kim et al.^55^ arranged symmetric fixed electrodes on both sides, enabling bi-directional tunability and offering further advantages in high-precision scenarios. These advancements broaden the application scope of MEMS inertial switches, particularly in situations requiring quantitative acceleration data.

### Enhanced contact and self-latching mechanism

The performance of MEMS inertial switches depends on critical parameters such as response time, contact duration, and factors affecting safety and reliability^15^. Among these, electrode contact quality is vital. Contact bouncing in fixed electrodes made of high-elasticity materials (e.g., silicon, gold, nickel) presents a significant challenge. This phenomenon makes it difficult to achieve adequate contact durations needed for circuit activation and data transmission^56^, compromising signal processing accuracy and switch reliability^57,58^. Furthermore, short contact times make it challenging to identify brief pulse widths and add to the complexity of CMOS designs for signal processing circuits. They can also blur the distinction between noise and actual switching signals, leading to false triggering^15,22,29,57–60^. Transient switching combined with mechanical hammering and electrical arcing increases the risk of electrode interface damage. Such interface degradation reduces system durability and risks permanent electrode adhesion^61^, particularly during multi-cycle testing, where switches degrade rapidly and often fail only after a few cycles^56^.

To improve signal reliability in high-performance MEMS inertial switches, enhancing contact quality is a common practice^57^. Considerable efforts have been devoted to exploring various strategies, including structural design, materials selection, and force control^58^. Flexible electrode structures and materials effectively reduce bouncing and extend contact duration. Cai et al.^22^ demonstrated improved stability using compliant cantilever stationary electrodes combined with dual mass-spring movable electrodes, allowing elastic deformation during contact (Fig. 1g). Lee et al.^62^ achieved 15-fold contact time extension (from 7.5 µs to 114 µs) through carbon nanotube (CNT) contact pads exploiting elastic deformation.

Traditional intermittent switches struggle with brief shock accelerations, which last only milliseconds. These switches reset after acceleration, which raises concerns about reliability in capturing dynamic shocks and notifying users effectively^63^. Latching mechanisms overcome this limitation by maintaining stable on-states, proving beneficial for accurate event capture and documentation^64^, particularly in applications with intermittent power sources or shock-triggered activations. Mechanical latching switches guarantee continuous activation upon shock detection, simplifying signal processing, providing prompt alerts, and enhancing safety and inspection speed^63^. These switches eliminate constant power monitoring needs and resist electromagnetic disturbances, making them suitable in high-reliability systems such as airbags and rockets^27,55^.

Four implementation strategies facilitate self-latching functionalities: mechanical latches, bistable switches, liquid metals, and magnetic/electrostatic forces. Mechanical latches and bistable switches extend contact durations through mechanical deformation and interaction^51–53,65–71^. Lee et al.^54^ developed a MEMS switch maintaining on-state post 43.7 g acceleration (Fig. 1h). Liquid metal solutions like the microscale liquid-metal droplet used by Yoo et al.^72^ (Fig. 1i) can remain closed even if the acceleration load disappears or under vibration interferences^72–74^. However, the toxicity of certain liquid metals, such as mercury, along with their sensitivity to temperature fluctuations, raises concerns about safety and reliability. Magnetic/electrostatic systems provide tunable thresholds along with self-latching capabilities^63,65,75^. Zhang et al.^63^ extended contact time by increasing the bias voltage and enabled electrostatic latching once the critical bias voltage was reached. These developments underscore the need for contact quality enhancement and latching mechanism integration to advance MEMS switch reliability.

## Technology path for design objectives

**Fig. 2:**
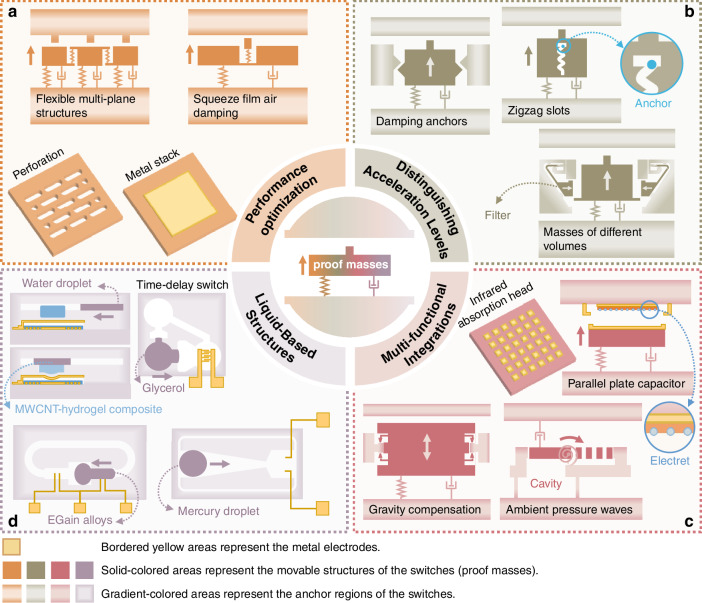
Sensitive structures are typically proof masses supported by springs, allowing movement in specific directions. **a** Squeeze film air damping^22,30^ and flexible multi-plane structures^76^ can prolong the contact time and eliminate rebounding. Perforation can mitigate structural warping^57^, and metal stacks can serve as movable electrodes^107^. **b** Damping anchors^70,78^ and zigzag slots^32^ enable differentiation between launch accelerations and accidental drops while utilizing masses of different volumes to achieve acceleration band-pass characteristics^71^. **c** Multiple functions can be integrated, including an infrared absorption head for sensing infrared signals^26,113,114^, ambient pressure wave sensing for responding to acoustic signals^81^, gravity compensation for achieving sub-g thresholds^79^, and a parallel plate capacitor for self-powered functionality^80^. **d** Liquid-based structures, such as MWCNT-hydrogel composites^84^, glycerol^82^, EGain alloys^83^, and mercury droplets^72,74^, can deliver excellent performance features, including no bounce, low resistance, high current capacity, and time-delay functionality

Critical to inertial switch operation, the mass, spring, electrodes, and actuators require coordinated design. These devices typically employ a spring-mass system (Fig. 2) where a suspended mass moves via precision springs. This configuration converts external acceleration into measurable displacement relative to fixed anchors, with sensitivity determined by mass-spring parameters. Two contact electrodes—one mobile on the mass and one fixed on the anchor—enable electrical switching (Fig. 4). Acceleration-induced mass movement opens/closes this contact pair, generating binary electrical signals. Integrated MEMS actuators provide precise mass control, allowing adjustable acceleration response thresholds. Optimizing these components ensures sensitivity, accuracy, and reliability. This section analyzes existing design methodologies and performance enhancements to guide future development.

### Sensitive structures

Advanced inertial switches integrate sensitive structures combining substantial proof masses with enhancement of reliability, intelligence, and functionality. Reliability improvements stem from structural innovations (Fig. 2a): Flexible connections that link the contact point to the mass enable squeeze-film air damping between mass and substrate to regulate frequency response^22^, maintaining ON-state duration under large accelerations through the squeeze-film effect of air in the narrow gap^30^. Multi-plane flexible structures reduce pull-in voltage and suppress rebound by breaking down the displacement into smaller, incremental steps across multiple electrodes^76^. Coating the proof mass with a Ti/Pt/Au or similar metal stack is widely adopted to enhance conductivity for improved current-carrying capacity^77^, while perforation patterns mitigate warping caused by electroforming-induced residual stress^57^

Smart acceleration discrimination employs damping mechanisms (Fig. 2b): Damping anchors with unique tooth profiles and pairs enable launch-phase acceleration recognition for artillery fuse safety^78^. Likewise, integrated zigzag slots with an anchor inside differentiate launch accelerations from accidental dropping through motion damping^32^. Furthermore, band-pass filtering via filter masses blocks switch activation at high accelerations, achieving acceleration band-pass characteristics^71^.

Specialized mass design enables multifunctional enhancement(Fig. 2c): Gravity-assisted electrode gap modulation achieves ultra-low threshold compensation^79^. Capacitive mass-electret configurations create self-powered switches^80^. Acoustic sensing is realized through pressure wave-induced torque generation in resonant masses^81^. Metal-insulator-metal plasmonic IR absorber arrays integrated with the mass structure enable infrared detection^3^.

Liquid-phase alternatives address solid-contact limitations (Fig. 2d): Traditional solid-to-solid contacts suffer from issues like wear, signal bounce, instability, and high contact resistance^73^. In contrast, liquid alternatives, such as liquid metal droplets^72,74^ or glycerol^82^, demonstrate no contact bounce, low contact resistance, and high current capacity^73,83^. Microfluidic switches made of multiwall carbon nanotube (MWCNT)-hydrogel composite activate via acceleration-driven water droplet movement^84^. Similarly, gallium-indium (EGain) alloys, characterized by non-toxicity, low viscosity, and high conductivity, enable tunable threshold acceleration, response time, and connection time through modifications in valve geometry and electrode positioning^83^. Despite these advantages, their practical implementation is constrained by short storage lifetimes and narrow operational temperature ranges^83^.

### Suspension structures

**Fig. 3:**
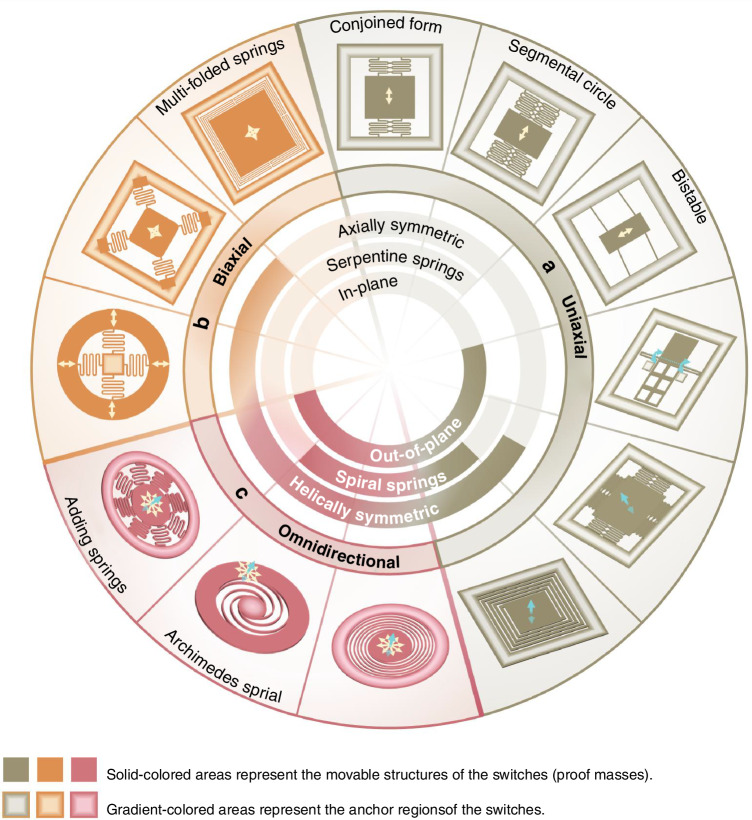
Suspension structure design. **a** Uniaxial switches in recent years often incorporate axially symmetric springs^24,25,59,63,66,81,86^ for enhanced stability and precise movement control. Many of these switches utilize serpentine springs^24,25,59,63,86^ to provide low stiffness. The sensitive direction of these switches (in-plane^25,59,66,86^, out-of-plane^24,63,77^, or rotational^81^) is determined by the size parameters and position of the springs. **b** In biaxial switches^28,39,115,116^, the springs are often designed with helical symmetry to ensure uniform compliance in two perpendicular in-plane directions. Serpentine springs are also popular in biaxial switches^28,115,116^. **c** In omnidirectional switches^29,42,44,93,107^, the suspension structure should homogenize the in-plane stiffness and ensure a comparable z-axis stiffness. Therefore, helically symmetric spiral springs^29,42,107^ are frequently employed to enhance stiffness uniformity, lower the threshold, and minimize footprint simultaneously

Suspension structures position the proof mass above the substrate^21,85^, enabling its acceleration-responsive motion while maintaining stability. Sensitivity improvements rely on low-stiffness designs that allow distinguishable proof mass displacements under acceleration. These structures are tailored to sensing directionality—uniaxial, biaxial, and omnidirectional (Fig. 3). To ensure further stability, symmetric layouts are frequently used to compensate for temperature variations and residual stresses for reliable operation^26^. Bi-stable designs further extend functionality beyond basic support roles.

MEMS uniaxial inertial switches optimize stiffness contrast between sensitive and non-sensitive axes, thereby enhancing sensitivity in a single axis while minimizing sensitivity to off-axis movements^58^. Axially symmetric springs (Fig. 3a) provide stability and precise control over proof mass motion, where paired conjoined springs replace four separate units, allowing for coordinated movement^59^. Segmental circular springs reduce directional stiffness compared to traditional semicircular springs^86^. Off-plane sensitivity suppression can be achieved via laterally driven switches with double-layer springs^87^, where symmetric spring distribution and constraint structure limit off-axis proof mass displacement under acceleration disturbances.

Multi-axial switches include in-plane (biaxial or two-dimensional omnidirectional) and three-dimensional variants (triaxial or three-dimensional omnidirectional). Most three-dimensional switches occupy a transitional category, combining in-plane omnidirectional motion with z-axis freedom. While two-dimensional omnidirectional designs inherently incorporate z-axis freedom through thickness adjustments, all three types—two-dimensional omnidirectional, triaxial, and three-dimensional omnidirectional—are grouped as omnidirectional in this review. Despite this classification, practical implementations remain distinct from idealized three-dimensional omnidirectional systems.

Multi-axial switches (Fig. 3b, c) position holding points at the proof mass center for mechanical balance^16,28,44,45^ and multi-directional motion. Four multi-folded springs enable bidirectional detection^39^, while helically symmetric spiral or serpentine springs homogenize in-plane stiffness. Both spring types minimize footprint while providing the desired sensitivity^22^. Archimedes’ spiral, for instance, offers omnidirectional compliance with reduced stiffness^16,88^. Increasing the number of springs effectively enhances stiffness uniformity, but too many springs may lead to overly high structural stiffness^44^.

MEMS micromachining enables in-plane flexibility for creating intricate two-dimensional geometries, but faces fundamental limitations in three-dimensional omnidirectional sensitivity: geometries are confined to two-dimensional planar definitions where thickness alone governs out-of-plane parameters. While parameter optimization can align z-axis stiffness with in-plane values, the intrinsic disparity between out-of-plane and in-plane bending behaviors prevents uniform stiffness across spherical directions^42^. Consequently, achieving consistent and reliable three-dimensional sensitivity remains an unresolved challenge.

Bistable beam suspensions (Fig. 3a) exploit the post-buckling mechanism to establish dual equilibrium states that store and release nonlinear elastic energy, creating a distinct threshold based on the structural characteristics and the onset of instability^58^. This enables latching behavior that maintains post-activation position and enhances contact reliability^66^. This design reduces friction and wear, bolstering overall durability and lifespan^89^. Due to its significantly lower snap-back acceleration than snap-through mechanisms, this design can retain its secondary stable state to achieve extremely low acceleration thresholds^90^. However, bistable switch design requires balancing numerous structural parameters—post-buckling geometry, inertial stress, snap distance, and activation forces, which complicate optimization^89^. Furthermore, fully axially constrained designs exhibit temperature and residual stress sensitivity, degrading performance and stability^58^. Batch fabrication inconsistencies also further compromise device reliability and production reproducibility^91^.

### Contact electrodes

MEMS inertial switches employ contact electrode pairs to convert proof mass displacement into a binary electrical signal or establish activation pathways. Contact duration critically influences both signal processing and circuit operation. Multiple contact pair configurations enable multi-directional or multi-threshold capabilities.

Contact gap minimization offers a practical approach to enhancing contact quality and lowering activation thresholds. Sub-micron electrode gaps effectively reduce required trigger signals, thereby improving sensitivity^19^. A diminished gap reduces the cantilever restoring forces, allowing contact adhesion forces to be large enough to prevent reopening after activation. This creates a “latched” state with memory functionality that persists until manually reset^26^. However, achieving smaller contact gaps requires more complex or costly processes, and inherent fabrication limitations make further reductions increasingly challenging and less stable.

**Fig. 4:**
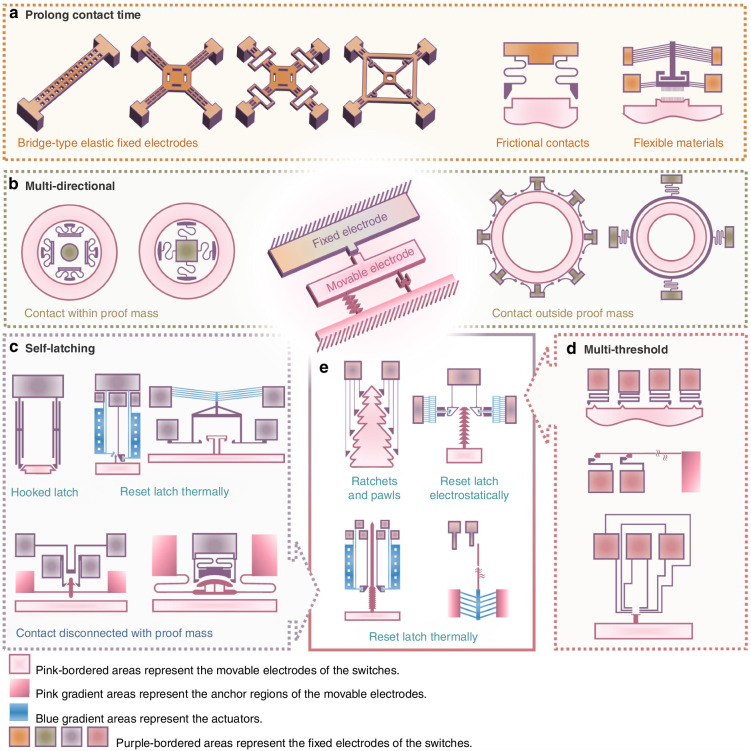
Contact electrode design. **a** Stiffness reduction^22,92,117,118^, frictional contacts^57^, and flexible materials^62,93^ have been identified as effective methods for prolonging contact time. **b** Achieving multi-directional capability can be completed using circularly symmetrical contacts, either within^46,88^ or outside the proof mass^28,96^. **c** The hooked latch^54^ is a typical structure for self-latching, which can be optimized by incorporating a thermally reset actuator^21,95^ or disconnecting it from the proof mass^27,70,78^. **d** Multi-threshold capability can be realized by distributing multiple electrodes along the sensing direction^47,49,97^. **e** Self-latching and multi-threshold capabilities can be integrated by incorporating multiple hooked latches or employing ratchets and pawls^51^. This integration can further include reset capabilities, either electrostatically^52^ or thermally^21,48^

Extended contact duration is crucial for reliable signal processing. Key approaches include reducing electrode stiffness, utilizing contact friction, and integrating flexible material (Fig. 4a). Spring buffers simultaneously protect against impact damage, suppress contact bouncing, and prolong contact duration^39,46^. Bridge-type elastic electrodes with holes have emerged as practical solutions for reducing stiffness and enhancing the contact effect and duration^59^. Strategic dimensional modifications to fixed electrodes (shape, thickness, width, and length) further optimize contact time^92^. Positioning the contact point at the proof mass center in bridge-type designs converts the distributed load into a concentrated one, enhancing contact efficacy and prolonging activation^23^.

Frictional contacts and flexible materials provide complementary solutions to transient contact durations and bouncing issues. Frictional designs dissipate kinetic energy through interface resistance, stabilizing contacts while extending contact durations without rebound^57^. Flexible materials like carbon nanotubes (CNTs)^62,93^ and polymer-metal composites^44^ provide alternative solutions through mechanical flexibility and resilience that enhance contact performance. CNT implementations exploit nanotube elasticity and inter-pad frictional adsorption to sustain prolonged contact times^15^. However, these materials can be challenging to fabricate and may be prone to structural damage or delamination when subjected to mechanical shocks^58^.

Latching mechanisms maintain persistent contact stabilization post-activation, ensuring an indefinite contact time^32,35,71,94^. These mechanisms typically employ hooked latch configurations (Fig. 4c) with electrostatic, thermal, or mechanical resets for reusability^15,21,48,70,91,95^. By isolating contact from the proof mass vibrations, these mechanisms improve operational reliability^27^. MEMS inertial switches can be customized for multiple thresholds or sensitivity along multiple axes by arranging fixed electrodes in different configurations (Fig. 4b, d). Orthogonal electrode configurations permit biaxial, triaxial, or omnidirectional detection. Discrete electrode placement along sensitive axes establishes multiple acceleration thresholds^28,46,47,49,88,96,97^. Combining multi-threshold capabilities with latching functionality enables shock-level-dependent triggering (Fig. 4e), yielding reliable quantitative acceleration data through multi-threshold latching^21,47,48,50,52,53,98^.

### Actuators

**Fig. 5:**
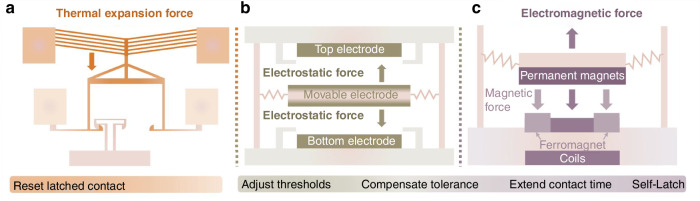
Actuator design. **a** The thermal actuator utilizes thermal expansion force to reset latched contact^95^. **b** The electrostatic actuator employs force to adjust thresholds, compensate for tolerance, extend contact time, and achieve the latch function^41^. **c** The electromagnetic actuator^65^ uses magnetic force to perform analogous tasks

MEMS inertial switch design integrates thermal, electrostatic, and electromagnetic actuation mechanisms to enhance functionality (Fig. 5). While thermal actuators exhibit high power consumption, their large output forces make them practical for resetting latching structures. For instance, infrared sensors employ buried microheaters to thermally actuate bottom contacts and restore open states post-detection^26,99^. Similarly, Currano et al.^95^ developed V-beam-style thermal actuators as shock sensor reset mechanisms (Fig. 5a), activated via applied current.

Electrostatic actuators are popular due to CMOS compatibility and low power requirements^100^. These systems utilize paired capacitive electrodes on contact or sensitive structure (Fig. 5b). Applied bias voltages reduce contact separation, lowering energy thresholds for closure and enabling amplification function^19^. Combined with dynamic shock forces, this configuration decreases activation acceleration thresholds^47,64^ while allowing voltage-based threshold tuning^101^. Such post-fabrication adjustability compensates for fabrication deviations^41,64,85,99^ and enables user-programmability settings^21^. Electrostatic latching mechanisms can also maintain stable on-states after the shock has subsided, with voltage removal restoring off-states^63,64,75,101^.

Despite their usefulness, electrostatic systems require elevated voltages and stringent electrode surfaces compared to standard integrated circuits, increasing power demands and fabrication complexity. In contrast, electromagnetic actuation (Fig. 5c) offers reduced voltage needs, less stringent surface requirements, and inherent bidirectional capability^65^. Electromagnetic forces can be reversed by adjusting the current direction, surpassing electrostatic unidirectionality. Magnetic latching eliminates continuous voltage application, permitting manual coil-current resets^65^. However, using permanent magnets in electromagnetic actuation may increase the overall volume of devices. Furthermore, both actuation methods face limitations, including persistent power needs for threshold adjustment and electromagnetic noise susceptibility^58^.

### Fabrication processes

**Fig. 6:**
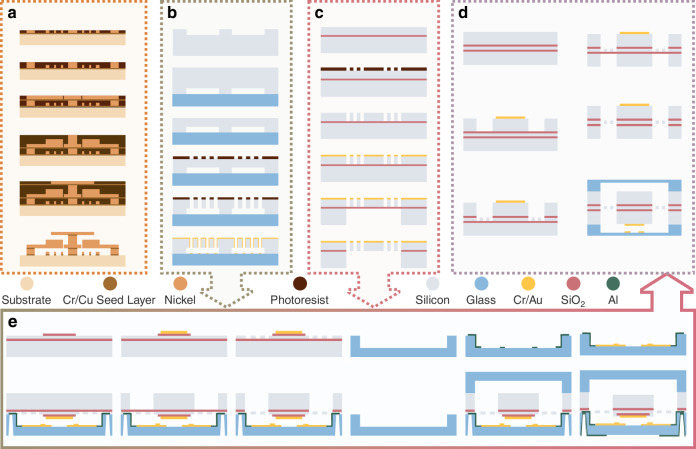
Fabrication processes design. **a** Surface process^44^. **b** SOG process^39^. **c** SOI process^21,81^. **d** Symmetric double-buried oxide SOI^107^. **e** Combining SOI and SOG processes^106^

The methodology for fabricating MEMS inertial switches highly depends on structural design and application scenarios, with bulk silicon and surface micromachining representing the dominant approaches. Multi-layer surface micromachining has emerged as a cost-effective alternative to silicon-based methods^91^, employing metal electroplating on insulated quartz substrates to create switch components (Fig. 6a)^22,23,25,38,44–46,57,59,66,86,88,102–104^. The proof mass thickness can be adjusted by controlling electroplating duration, increasing mass for greater inertial force and sensitivity under acceleration^59^. Typical surface-machined designs feature thicker proof masses than springs to minimize footprint and optimize sensitivity^22^. Nickel dominates as the structural material due to its silicon-comparable Young’s modulus and fourfold higher density, which improves mass-to-area ratios for threshold reduction. Nickel’s inherent conductivity further eliminates sidewall metallization needs^105^.

Surface micromachining faces challenges in low-g (<30 g) switches fabrication, where large proof masses and multi-step electroplating exacerbate residual stress and threshold inaccuracies^61,91^. Bulk silicon methods like Silicon-On-Glass (SOG)^28,39,60,61,101^ and Silicon-On-Insulator (SOI)^21,43,47^ techniques (Fig. 6b, c) provide superior uniformity. The SOI process enables precise single-crystal silicon device layers that reduce threshold variations caused by fabrication deviations. Integrating SOI and SOG processes allows for thicker proof masses than springs in silicon bulk micromachining. As demonstrated in Fig. 6e, the device layer fabricates the springs, while both the device and handle layers form the proof mass^106^. Symmetric double-buried oxide SOI wafers (Fig. 6d) further address mechanical imbalance and tilt issues from off-center springs^29,77,107^. These silicon-based approaches require contact metallization to compensate for the poor conductivity of silicon.

Fabrication technology is influenced by the intrinsic properties of metal and silicon. Metallic switches enable omnidirectional sensitivity through superior conductivity and overload resistance. However, they suffer from thermal expansion-induced threshold variations, limiting their use in wide-temperature environments like missile-equipped devices. Single-crystalline silicon (SCS) has a low coefficient of thermal expansion^106^, making it suitable for such environments despite its inherent fragility and poor conductivity. The selection of materials and fabrication processes thus becomes dictated by device structure, cost, threshold, accuracy requirements, and operational conditions.

## Applications of MEMS inertial switches

**Fig. 7:**
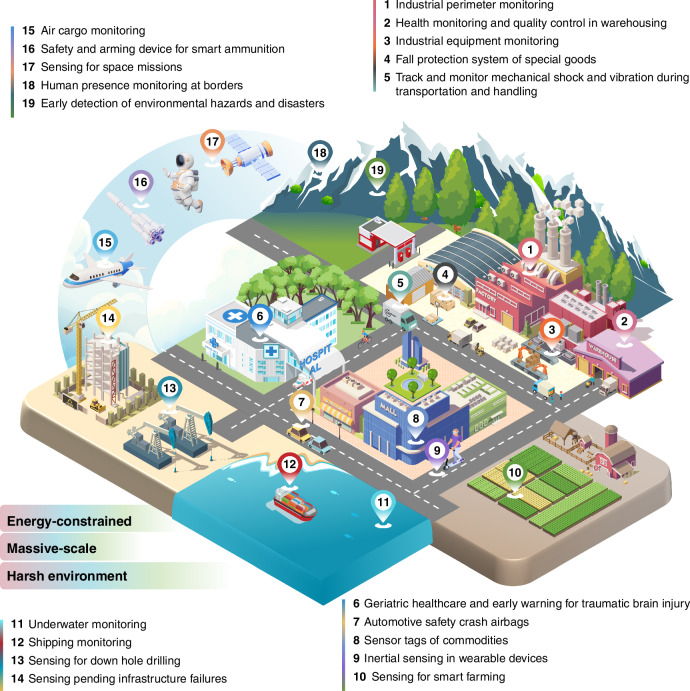
Applications of MEMS inertial switches

The rapid development of MEMS inertial switches has led to impressive features, including small size, low power consumption, minimal data output, affordability, high sensitivity, excellent impact resistance, multi-functionality, and intelligence. The potential applications of MEMS inertial switches are extensively researched across numerous fields, particularly in the IoT and embodied intelligence that operate in conditions with limited energy, massive scale, or harsh environments, as shown in Fig. 7.

### Energy-constrained

MEMS inertial switches remain dormant until activated by threshold acceleration events, requiring zero standby power. Commercial accelerometer systems exhibit non-negligible persistent energy draw—wireless structural monitoring boards consume 70–750 mW during operation from active electronic components (ADCs, MCUs, and transmitters)^108^, limiting battery lifespan to six months even at <7% duty cycles. MEMS inertial switches overcome such limitations through mechanical integration of sensing, threshold comparison, and data storage, achieving zero static power by eliminating electronic components. Their standby mode operates below 10nW, extending operational lifetimes to multiple years in unattended deployments^4^.

This inherent energy efficiency renders them suitable for deployment in maintenance-prohibitive environments, including underwater systems, remote borders, and space. It enables deploy-and-forget border security and perimeter monitoring networks that detect intrusions and environmental disasters^18,19^. Its compact size and cost-effectiveness further support covert, energy-limited, and high-granularity sensor networks requiring maintenance-free long-term operation. Missile health monitoring systems similarly require MEMS wake-up switches to predict post-storage performance and identify potentially damaged units^21^ while adhering to strict energy and space constraints. In healthcare, MEMS inertial switches eliminate the need for continuous power connections or frequent recharging, making them particularly valuable in wearables systems and geriatric care. For example, smart shirts integrated with MEMS inertial switches can detect falls, activating alarms to trigger air-inflatable hip protectors or alert caregivers^20,22,23^, thereby minimizing injury risks. These devices also enhance medical rescue efficiency by providing timely injury feedback^57^, with notable applications in traumatic brain injury (TBI) early-warning systems^42^.

### Massive-scale

While energy supply proves feasible in some applications, reliably powering distributed sensors or sustaining battery replacements remains challenging and cost-prohibitive for large-scale monitoring systems in infrastructure, smart agriculture, industrial, and logistics sectors. These systems further require substantial computational resources for managing massive datasets. Integrating ever-alert battery-less MEMS inertial switches into remote sensing systems like RFID sensor tags alleviates these issues^26^, enabling cost-effective wireless monitoring of diverse assets at unprecedented scales.

Globalization-driven logistics expansion necessitates damage prevention through goods monitoring during delivery, unloading, and distribution. Acceleration monitoring becomes critical for fragile items, as mechanical shocks increase damage risk^109^. MEMS inertial switches emerge as the preferred solution due to their small size, high sensitivity, and cost-effectiveness^63^. Embedded within products, packaging, and shipping containers, these devices enable remote monitoring of mechanical shock and vibration, surpassing conventional visual shock indicators^21,23^. Acceleration data transmitted via radio-frequency components facilitates damage risk assessment and lifecycle tracking of goods, including structural integrity, maintenance requirements, and operational longevity^21,22^.

Wireless inertial switches enable real-time threshold acceleration monitoring for remote crash and drop protection systems^66^. They can activate more sophisticated sensing and safety mechanisms from low-power sleep states, allowing for environmental data collection and protection against severe damage from sudden impacts^21,66,72^. The emergence of advanced ultra-low threshold MEMS inertial switches (<1 g detection) significantly enhances protection for fragile goods and portable electronics during free-fall events^15^.

### Harsh environment

The aerospace, defense, and energy sectors increasingly require rugged MEMS inertial switches capable of enduring extreme conditions^31^. These applications demand sensors that function under thermal extremes, intense vibrations, and radiation exposure, while smart ammunition requires survival through strong shock exceeding 10,000 g from gunfire. Marozau et al.^110^ evaluated three commercial MEMS accelerometers under simulated space mission conditions, revealing critical reliability issues in peripheral components rather than MEMS microstructures. Mechanical shock testing with 5000 g/0.2 ms pulses demonstrated consistent failures due to wire-bond separation from contact pads in packaging, with no observed MEMS structural damage. Temperature cycling (−65 °C to +185 °C) similarly identified interconnect wire debonding as the dominant failure mechanism. Radiation exposure studies (110 Krad gamma/100 Krad proton) confirmed no mechanical damage to MEMS structures or wire bonds. Failures were primarily attributed to ASIC degradation caused by gamma and proton irradiation.

MEMS switches, with their minimal reliance on wire bonds and ASICs, demonstrate superior resilience in extreme environments. Their non-electronic operational principles inherently resist electromagnetic interference, radiation, thermal stress, and mechanical impacts. For example, Guo et al.’s high-G switch withstands 15,000 g shocks using arch truss fracture mechanisms and proof-mass-independent contact paths^34^. Sun et al. developed a thermally stable switch with a buckle-beam structure that exhibits less pull-in voltage sensitivity to temperature (−20 °C to 100 °C) and maintains <1.5% variation post-annealing at 280 °C for 12 h^111^.

Military modernization drives MEMS integration into advanced weapon systems, particularly for miniaturized safety mechanisms in missiles and smart weapons^101,103^. MEMS inertial switches enable critical functions in missile applications through micrometer-scale dimensions and nanowatt-level power consumption, activating when exceeding predefined acceleration thresholds. These capabilities prove essential for ignition safety devices (ISDs), safety-arming (S-A) devices, and fuses in missiles^78,82,106^. Lee et al.^112^ reported an S-A switch for the rocket motor ignition system in cold-launched missiles. The inertial switch activates at ~10 g acceleration along its sensitive axis while resisting unintended hundreds-g impacts in off-axis directions, ensuring reliability in harsh military environments. Projected automobile market growth will also increase demand for MEMS inertial switches, particularly in crash airbag safety systems^103^. These switches reliably detect accelerations to activate vehicle safety mechanisms during collisions^29,30^, while minimizing false triggers from electromagnetic noise^22,23^. Their operational robustness makes them critical components for advancing automotive safety.

## Outlook

MEMS inertial switches are event-based sensors with micrometer-scale dimensions and nanowatt-level power consumption. These features enable energy-efficient and resource-conserving applications, particularly for IoT and embodied intelligence systems. Despite their promising potential, three primary challenges currently hinder their widespread implementation:

### Universality

Fixed-threshold operation limits adaptability across diverse application scenarios. Customization through size parameters and structural modifications diminishes deployment efficiency and escalates production costs.

### Low threshold

Reliable low-threshold switches, particularly below 1 g, remain problematic due to designs requiring large proof masses and flexible suspensions. These configurations are prone to mechanical damage and sensitive to fabrication deviations, internal stresses, and thermal fluctuations.

### System integration

Current research emphasizes switch design with limited exploration of their integration within broader microsystems. Practical implementation requires efficient integration with alarm and processor systems, which is crucial but underdeveloped.

To tackle the challenges, ongoing research efforts are concentrated on designing multiple contact electrodes, incorporating built-in actuators, and optimizing contact performance. While promising, these approaches face inherent limitations that necessitate continuous innovation and iterative refinement.

### Multiple contact electrodes

Multi-electrode designs improve adaptability through discrete thresholds. However, large threshold disparities can compromise precision, whereas minimal differences restrict versatility. Moreover, implementing multiple thresholds within a single device increases design complexity, enlarges the device footprint, and raises data processing demands. Hence, future research should focus on innovative mechanical structures and simplified data-reading methods.

### Actuator integration

Electrostatic and electromagnetic actuators provide external forces for adjustable thresholds and enable the realization of low thresholds. However, electromagnetic actuators hinder miniaturization, while electrostatic actuators require continuous high-voltage circuits. This leads to power consumption in the control and boost circuit that contradicts the zero power objective during dormancy. Additionally, both actuators are susceptible to external electromagnetic interference, potentially causing false triggering and reducing reliability. Therefore, developing more stable and energy-efficient methods for threshold adjustment is imperative.

### Contact enhancement

Incorporating contact enhancement and self-latching mechanisms improves the reliability of triggering signals and electrical pathways, facilitating easier system integration. However, embodied intelligence applications face challenges due to the shift from static frames to event streams in SNNs. This shift calls for developing ad hoc interfaces, communication protocols, and event-handling software, along with clearly defined parameters, such as response time and current-carrying capacity, to guide switch design.

Advancing structural designs, actuation mechanisms, and system integration is essential to fully leverage MEMS inertial switches’ advantages in energy-constrained, large-scale applications. These switches offer a compelling alternative to traditional battery-powered sensors, particularly in the burgeoning landscape of IoT and embodied intelligence systems. Their sparse data output and ultra-low power consumption enable strategic resource allocation in IoT networks, supporting real-time, adaptive monitoring and control. The motivation, approaches, and purposes outlined in this review are expected to contribute to the development and broader adoption of MEMS inertial switches, enhancing their performance and streamlining their integration into IoT and embodied intelligence applications. Ultimately, this progress brings us closer to creating more efficient, responsive, and sustainable interactions with the physical world.
